# Metabolite profiling during graft union formation reveals the reprogramming of primary metabolism and the induction of stilbene synthesis at the graft interface in grapevine

**DOI:** 10.1186/s12870-019-2055-9

**Published:** 2019-12-30

**Authors:** Duyên Prodhomme, Josep Valls Fonayet, Cyril Hévin, Céline Franc, Ghislaine Hilbert, Gilles de Revel, Tristan Richard, Nathalie Ollat, Sarah Jane Cookson

**Affiliations:** 1 INRA, Univ. Bordeaux, ISVV, EGFV UMR 1287, F-33140 Villenave d’Ornon, France; 2 Unité de recherche Oenologie, EA 4577, USC 1366 INRA, ISVV, Université de Bordeaux, F33882 Villenave d’Ornon, France

**Keywords:** Grafting, Grapevine, Scion, Rootstock, Canes, Wood, Stilbenes, Flavanols, Sugars, Amino acids

## Abstract

**Background:**

Grafting with rootstocks is essential for the culture of many perennial fruit crops and is increasing being used in the production of annual fruits and vegetables. Our previous work based on microarrays showed that transcripts encoding enzymes of both primary and secondary metabolism were differentially expressed during graft union formation in both homo-grafts (a genotype grafted with itself) and hetero-grafts (two different genotypes grafted together). The aim of this study was to profile primary and secondary metabolites, and quantify the activity of phenylalanine ammonia lyase (PAL) and neutral invertase (NI) in the scion and rootstock tissues and the graft interface of homo and hetero-grafts of grapevine 1 month after grafting. Table-top grafting was done on over-wintering stems (canes) of grapevine and the graft interface tissues (containing some woody stem tissues and callus) were compared to the surrounding rootstock and scion tissues. The objective was to identify compounds involved in graft union formation and hetero-grafting responses.

**Results:**

A total of 54 compounds from primary and secondary metabolism (19 amino acids, five primary and 30 secondary compounds metabolites) and the activity of two enzymes were measured. The graft interface was associated with an increase in the accumulation of the branched-chain amino acids, basic amino acids, certain stilbene compounds and higher PAL and NI activity in comparison to the surrounding woody stem tissues. Some amino acids and stilbenes were identified as being accumulated differently between the graft interfaces of the scion/rootstock combinations in a manner which was unrelated to their concentrations in the surrounding woody stem tissues.

**Conclusions:**

This study revealed the modification of primary metabolism to support callus cell formation and the stimulation of stilbene synthesis at the graft interface, and how these processes are modified by hetero-grafting. Knowledge of the metabolites and/or enzymes required for successful graft union formation offer us the potential to identify markers that could be used by nurseries and researchers for selection and breeding purposes.

## Background

Grafting is widely used in horticulture because rootstocks can provide resistance to soil borne pathogens and abiotic stresses as well as to influence scion growth and performance [1]. Grafting uses the innate wound healing mechanisms of plants to join two different species together for agronomic interest. Graft union formation begins with the formation of a necrotic layer and adhesion between the two grafted partners; this is followed by the proliferation of callus tissue and the formation of functional xylem and phloem connections between the scion and rootstock [2]. Grafting is a considerable stress to plants and triggers wounding responses such as the production of reactive oxygen species [3], the expression of defense related genes [4–6], the accumulation of defense related and antioxidant enzymes [3, 7, 8], phenolic compounds [9] and the production of wound-induced callus [10].

Because of its agronomic importance, most studies of graft union formation focus on incompatibility responses, related to either poor graft-take soon after grafting or delayed incompatibility responses occurring months to years after grafting. Accumulation of certain secondary metabolites at the graft interface has been observed in incompatibility responses in fruit trees [9, 11], olive trees [12], eucalyptus trees [13] and in the case of dieback of grapevine variety Syrah [14, 15]. Different sampling strategies have been used (sampling above versus below the graft interface, at the graft interface, in bulk tissue or isolated phloem) and samples have been taken at different times after grafting (from days to many years). Although the accumulation of some flavonoids has been already reported in various tissues and scion/rootstock combinations of grapevine [14, 15], to date there have been no reports on the accumulation of stilbenes at the graft interface in any species. Stilbenes are a small family of secondary metabolites found in a number of unrelated families including *Vitaceae*. Stilbenes are accumulated in grapevine tissues in response to a number of abiotic and biotic stress treatments, and are thought to protect against oxidative stress in addition to being phytoalexins [16, 17]. In addition to the accumulation of metabolites, the induction of the expression of transcripts encoding *PHENYLALANINE AMMONIA LYASE* (*PAL*), first and committed step in the phenyl propanoid pathway, has been associated with incompatibility responses in Prunus *spp.* [18, 19] and rubber trees [20], yet the activity of this enzyme was not measured. Increased PAL activity at the graft interface has the potential to increase the production of stress-related secondary metabolites with antioxidant functions well as providing precursors for the synthesis of lignin (necessary for xylem formation).

We have previously characterized the changes in transcript abundance at the graft interface of compatible homo-grafts [21] and hetero-grafts of grapevine [4]. The abundance of 22 transcripts putatively involved in the synthesis of secondary metabolites increased at the graft interface in a homo-graft 28 d after grafting; including 11 of the 35 stilbene synthases on chromosome 16 that were present on the microarray used [21]. In addition, we compared the transcripts differentially expressed at the graft interface between homo- and hetero-grafts of grapevine 28 d after grafting: 45 genes putatively involved in the synthesis of secondary metabolites were up-regulated at the graft interface of hetero-grafts, but no stilbene synthase genes [4]. This gene expression data suggests that the production of secondary metabolites may be important for graft union formation and hetero-grafting responses in grapevine. The aim of this study was to profile primary and secondary metabolites, and quantify the activity of PAL (EC.4.3.1.24) and NI (EC.3.2.1.26) in the scion and rootstock tissues and at the graft interface of homo and hetero-grafts of grapevine 1 month after grafting. A total of 54 compounds from primary and secondary metabolism (19 amino acids, five primary metabolites and 30 secondary metabolites) and two enzyme activities were measured using high-performance liquid chromatography, liquid chromatography coupled to mass spectrometry and enzymatic assays. .

## Results

### Primary metabolite profile of the rootstock, scion and graft interface tissue of woody grafts of grapevine

**Fig. 1:**
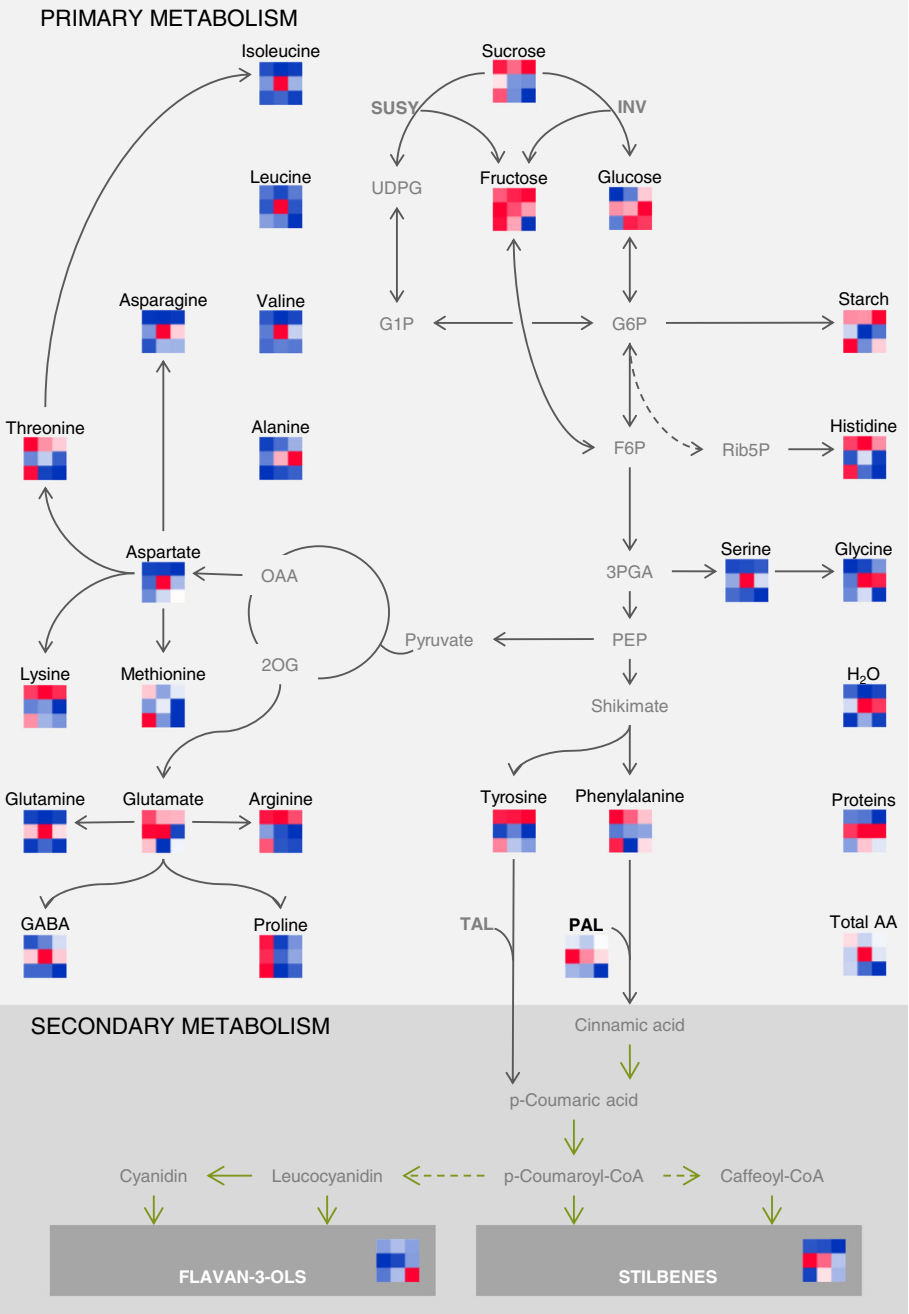
Simplified representation of primary and secondary metabolism showing heat maps of mean metabolite contents and enzymatic activities 28 d after grafting; red or blue indicate higher or lower metabolite content or enzymatic activity respectively. The first column shows the data from *Vitis vinifera* cv. Cabernet Sauvignon homo-grafts (CS/CS), the second and third columns show the data from CS grafted with the rootstocks *V. riparia* cv. Gloire de Montpellier and the *V. berlandieri* x *V. rupestris hybrid* cv. 1103 Paulsen respectively The first, second and third rows show the data from the scion, graft interface and rootstock respectively

The proliferation of callus tissues at the graft interface of the homograft *V. vinifera* cv. Cabernet Sauvignon (CS/CS) 28 d after grafting showed a distinct primary metabolite profile in comparison with the scion and rootstock tissues. This graft interface tissue (which contained both woody scion and rootstock tissues, and newly formed callus cells) had higher water, protein and Glc contents, and a lower concentration of starch compared to the surrounding tissues (Fig. 1., Additional file 1: Table S1). The water content is approximately 60% in the graft interface tissues and 50% in the scion and rootstock tissues. This high percentage of water is accompanied by the accumulation of proteins (about 3 mg g^− 1^ FW) in the graft interface tissue while in the scion and rootstock, the protein content was approximatively 1.7 mg g^− 1^ FW. Glucose concentration was higher in the graft interface (3 nmol g^− 1^ FW) in comparison with the scion and rootstock tissues (2 nmol g^− 1^ FW). Fructose accumulated slightly at the interface, but the difference was not significant. On the contrary, there is almost two times less starch in graft interface compared with the scion and rootstock tissues. The total concentration of amino acids did not differ between the three tissues studied, but the concentration of some individual amino acids was different between the graft interface and surrounding tissues. In particular, Gln and GABA contents were significantly higher, while Arg, Lys, His, Phe and Tyr contents were lower in the interface samples of CS/CS compared to the woody tissues of scion and rootstock.

The concentration of certain primary metabolites varied between the different rootstock wood samples studied; the concentration of starch, Fru, Suc, Arg, Pro, Phe, Thr, His and Met was higher in CS than either both or one of the American rootstock genotypes (*V. berlandieri* x *V. rupestris* cv. 1103 Paulsen (1103P) and *V. riparia* cv. Gloire de Montpellier (RG) (Fig. 1, Additional file 2: Table S2). However, the concentration of Glc was higher in RG and 1103P (Fig. 1, Additional file 2: Table S2). The concentration of Asn was two-fold higher in the wood of 1103P and the water content in RG (about 58%) was higher than in the two other genotypes (about 53%).

The graft interface samples of CS/CS had a higher starch and lower water content than those of CS/RG and CS/1103P (Fig. 1, Additional file 3: Table S3). Total amino acid concentration at the graft interface was highest for CS/RG and some individual amino acids were specifically accumulated at the graft interface in a manner which was unrelated to their rootstock tissue concentrations such as the accumulation of Asp, Ile, Leu, Val, Ser, Asn and Gln at the graft interface of CS/RG. Glycine was accumulated to higher concentrations in CS/RG and CS/1103P than CS/CS. The concentration of arginine at the graft interface reflected genotype-specific accumulation patterns in the rootstock wood.

The concentration of Pro was higher in all tissues of CS/CS than the two other scion rootstock combinations.

### Profiling the activities of PAL and NI at the graft interface, scion and rootstock tissues

In this study, PAL had a higher activity at the graft interface of CS/CS in comparison to the surrounding scion and rootstock woody tissues, NI was also increased, but the difference was not significant (Fig. 1, Additional file 1: Table S1). There were genotypic differences in NI activity in the rootstock wood (Fig. 1, Additional file 2: Table S2) and this was reflected in differences in NI activities in the graft interface tissues: NI activity was 10 nmol min^− 1^ g^− 1^ FW in CS/CS and CS/1103P, and 5 nmol min^− 1^ g^− 1^ FW in CS/RG (Fig. 1, Additional file 3: Table S3). However, there were no differences in PAL activity between the different scion/rootstock combinations (Fig. 1, Additional files 1: Table S1, Additional file 2: Table S2 and Additional file 3: Table S3).

### Profiling flavanol concentrations reveals a general decrease on flavanols at the graft interface

**Fig. 2:**
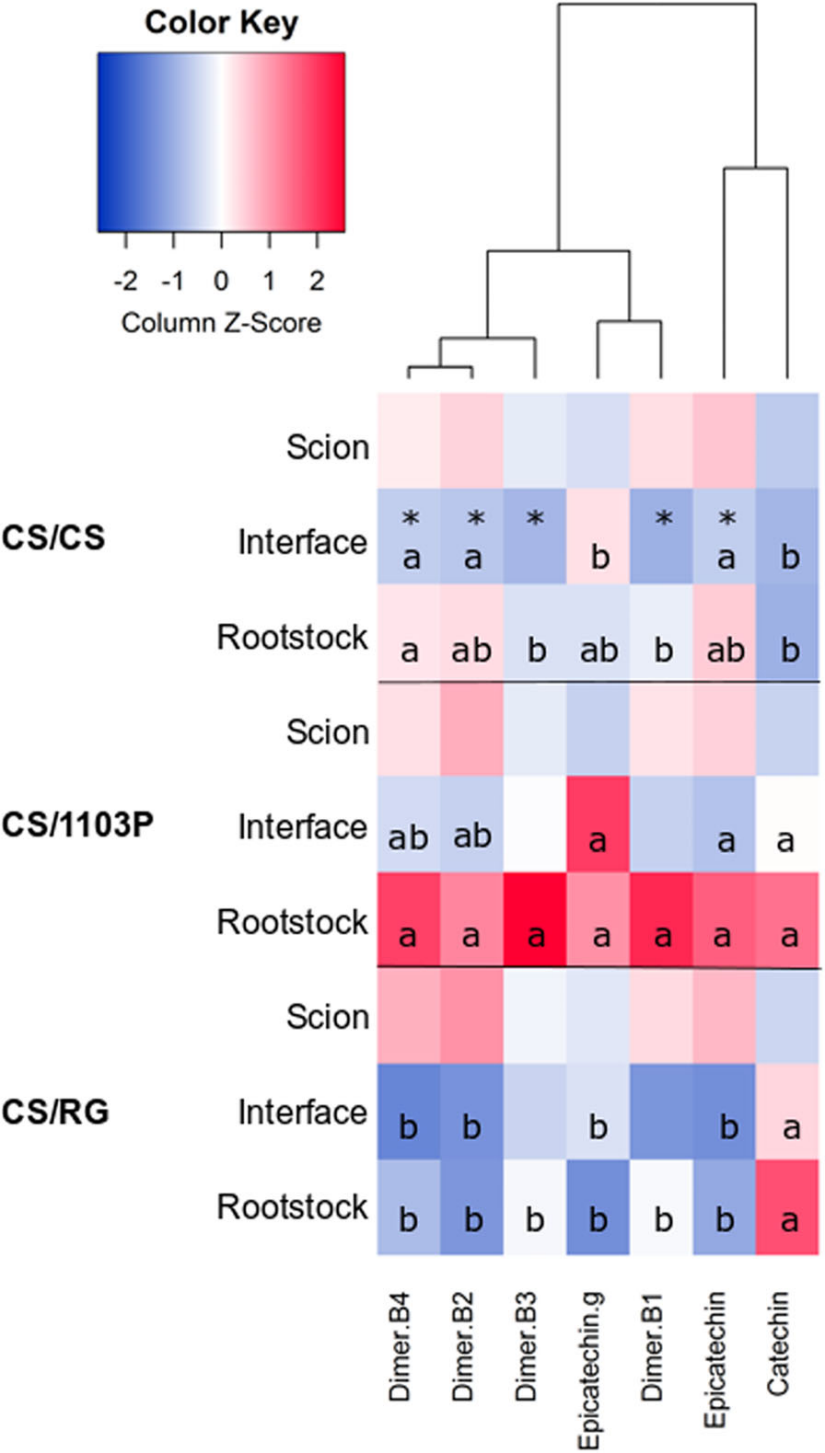
Heatmap of the mean concentration of flavanols in the scion, rootstock and graft interface tissues of *Vitis vinifera* cv. Cabernet Sauvignon homo-grafts (CS/CS) and CS grafted with the rootstocks *V. riparia* cv. Gloire de Montpellier (CS/RG) and the *V. berlandieri* x *V. rupestris* hybrid cv. 1103 Paulsen (CS/1103P) 28 d after grafting. Stars indicate the significant differences between the graft interface and scion and/or rootstock tissues of CS/CS (at the 5% level with either a one way ANOVA or Kruskal-Wallis test, Additional file 4: Table S4). The letters in the graft interface and rootstock data indicate the Tukey results of one way ANOVA or Kruskal-Wallis tests of flavanol concentration in the interface and rootstock (tested separately, Additional file 5: Table S5 and Additional file 6: Table S6 respectively)

Flavanols can exist in grapevine as monomers (catechin, epicatechin and their gallic acid esters) and as oligomers (which can also be called proanthocyanidins) [21]. Our analyses allowed us to identify several monomers and dimers (the total concentration of unidentified trimers was added to the total flavanol concentration data). Total flavanol concentration decreased at the graft interface of CS/CS (Fig. 2, Additional file 1: Table S1) and this decrease is primarily due to a decrease in the concentration of epicatechin as this was the most abundant flavanol present in the CS tissues (Additional file 4: Table S4). The rootstock wood tissues of the genotypes studied had different flavanol profiles; 1103P had a higher total concentration of flavanols than the two other genotypes (Fig. 2, Additional file 5: Table S5). The proportion of the different flavanols also varied between the genotypes; for example, there was a higher proportion of catechin in the rootstock samples of RG, whereas a higher proportion of the other flavanols in CS and 1103P (Additional file 5: Table S5). In general, differences in the concentration of flavanols at the graft interface were primarily due to differences in the concentrations of the metabolites in the rootstock wood (Fig. 2, Additional file 5: Table S5 and Additional file 6: Table S6).

### Stilbenes accumulate at the graft interface 28 days after grafting

**Fig. 3:**
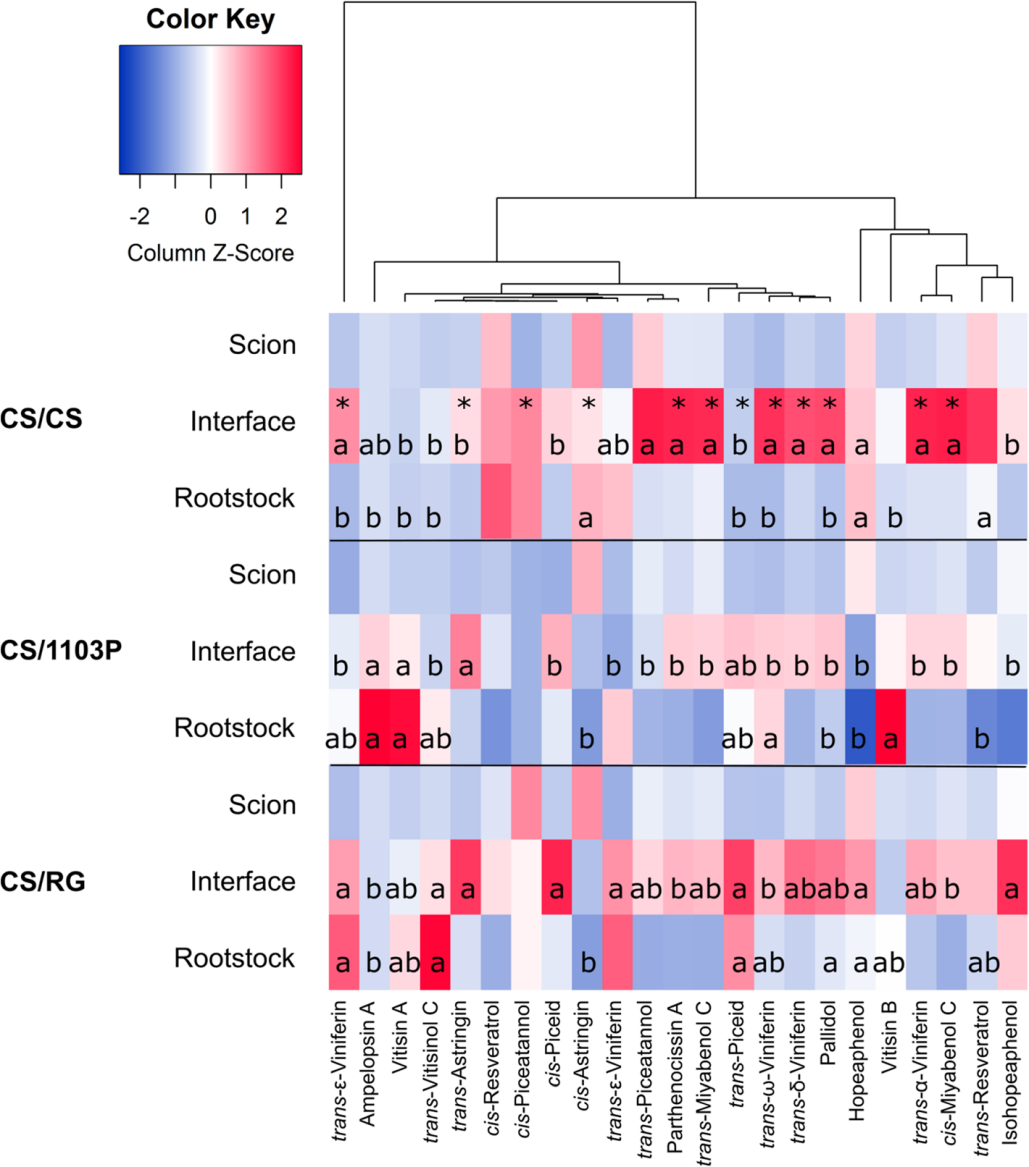
Heatmap of the mean concentration of stilbenes in the scion, rootstock and graft interface tissues of *Vitis vinifera* cv. Cabernet Sauvignon homo-grafts (CS/CS) and CS grafted with the rootstocks *V. riparia* cv. Gloire de Montpellier (CS/RG) and the *V. berlandieri* x *V. rupestris* hybrid cv. 1103 Paulsen (CS/1103P) 28 d after grafting. Stars indicate the significant differences between the graft interface and scion and/or rootstock tissues of CS/CS (at the 5% level with either a one way ANOVA or Kruskal-Wallis test, Additional file 7: Table S7). The letters in the interface and rootstock indicate the Tukey results of one way ANOVA or Kruskal-Wallis tests of stilbene concentration in the interface and rootstock (tested separately, Additional file 8: Table S8 and Additional file 9: Table S9 respectively)

The total concentration of stilbenes increased at the graft interface of CS/CS grafts 28 d after grafting; this was primarily due to an increase in the concentration of *trans*-ε-viniferin (the major stilbene found in CS wood, Fig. 3, Additional file 7: Table S7). In addition, other dimers and trimers such as *trans*-ω-viniferin, pallidol, *cis*-miyabenol C and α-viniferin increased over 4-fold in the interface compared to the rootstock or scion tissues (Fig. 3, Additional file 7: Table S7). Only one stilbene, *cis*-astringin, significantly decreased in concentration at the graft interface in comparison to the surrounding woody tissues.

### The profile of stilbenes in the rootstock wood is genotype-specific

The three genotypes of grapevine studied, CS, RG and 1103P, had different stilbene profiles in the rootstock wood tissues (Fig. 3, Additional file 8: Table S8), but the total stilbene concentration was not different between the genotypes (Additional file 2: Table S2). CS was richer in monomers and tetramers (mainly hopeaphenol), while RG and 1103P had higher concentrations of dimers. The concentration of the major stilbene present in the tissues, *trans*-ε-viniferin, was particularly high in RG. 1103P was rich in the tetramer vitisin A and an ampelopsin A dimer.

### Most stilbenes were differently accumulated at the graft interface of the different scion/rootstock combinations

The concentration of stilbenes at the graft interface of CS/CS and CS/RG was considerably higher than that of CS/1103P (Additional file 3: Table S3), and most individual stilbenes measured were higher in either CS/CS and/or CS/RG than in CS/1103P (except for ampelopsin A, which is at high concentrations in 1103P) (Additional file 9: Table S9). The concentration of stilbenes at the graft interface was generally associated with genotype-specific differences in the stilbene concentration in the woody tissues. However, it is noteworthy to mention that the particularly high concentration of ε-viniferin, pallidol and ω-viniferin at the graft interface of CS/CS were unrelated to genotype specific differences in the rootstock wood tissues. The increase of total stilbenes content at the graft interface relative to the rootstock wood was lower in both hetero-grafts than in the homo-graft controls. In both hetero-grafts, only a minor dimer (parthenocissin A), some trimers (*trans*- and *cis*-miyabenol C and α-viniferin), one tetramer (isohopeaphenol) and two monomers (*trans*- and *cis*-astringin) were highly increased at the graft interface.

### Principle component analysis (PCA)

**Fig. 4:**
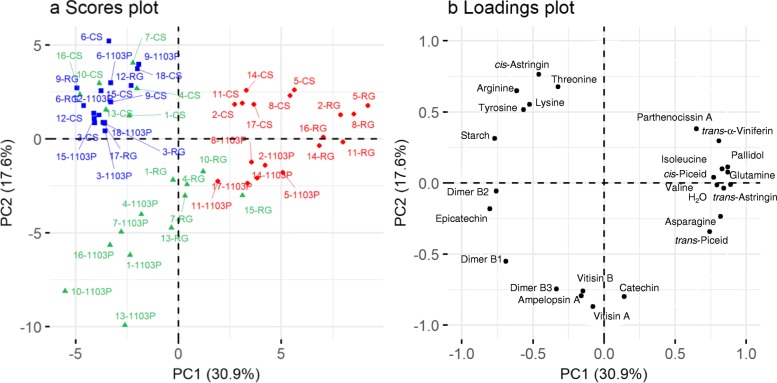
Principle component (PC) analysis of the key metabolites measured in the scion (blue), graft interface (red) and rootstock (green), **a** scores plot of the projection of individual observations on PC1 and PC2, and **b** variable loading plot

A PCA was done on all the variables measured in the scion, rootstock and graft interface tissues of the three scion/rootstock genotypes studied and to identify variables associated with either the tissue types or genotypes. The first two principal components (PCs) accounted for 49% of the total variance, with 31% explained by PC1 and 18% by PC2 (Fig. 4). In the scores plot, the PCA of the data shows that the samples clearly separate along PC1 according to the tissue type studied (Fig. 4a), with the woody samples of scion and rootstock on the negative side and the graft interface tissues on the positive side. The graft interface is associated with the accumulation of the amino acids Asn, Ile, Val and Gln, increased water content and the accumulation of the stilbenes *trans-* and *cis*-piceid, *trans*-astringin, pallidol, parthenocissin A and α-viniferin (Fig. 4b). Whereas the scion and rootstock samples were associated with high concentrations of epicatechin, the flavanol dimers B1 and B2 along with starch. Principle component 2 separates the samples according to the genotype studied (Fig. 4a), with CS being associated with high concentrations of Arg, Thr, Lys, Tyr, *cis*-astringin, and 1103P being associated with high concentrations of vitisin A and B, ampelopsin A, catechin and the flavanol dimer B3 (Fig. 4b).

## Discussion

### Graft union formation reprograms primary metabolism to support the proliferation of callus cells

Callus cells proliferate in response to wounding in most plant tissues [22] and at the graft interface as grafting employs intrinsic wound responses to join different genotypes together for horticultural interest. As the graft interface tissues contain young, rapidly dividing callus cells, these samples are richer in proteins and water than the surrounding woody tissues. Starch concentrations are reduced at the graft interface, while Glc accumulates. The accumulation of Glc at the graft interface coincides with the up-regulation of transcription of three vacuolar invertases and one hexose transporter [21]. There was a small increase in the activity of neutral invertase at the graft interface, although it was not significant. In perennial crops, grafting is typically done on overwintering, dormant plant material in the spring so that graft union formation coincides with the spring activation of growth [21]. Starch reserves in perennial structures are mobilized in the spring time to sugars to support growth at bud break [23, 24]; presumably starch is similarly mobilized to provide sugars to callus cell development at the graft interface. Similarly, Arg is the winter storage amino acid in woody tissues of grapevine [25] and it accumulated at high concentrations in the scion and rootstock. It is likely that Arg is mobilized and converted into others amino acids (particularly Gln) at the graft interface to support callus cell development. Glutamine is the most abundant amino acid in the graft interface tissues (and most plant tissues) and is the primary product of nitrogen assimilation from inorganic nitrogen sources and is therefore important for protein and nucleotide synthesis. The concentration of GABA was also high at the graft interface of the homo-graft CS/CS. γ-aminobutyric acid is synthesized from Gln via Glu and accumulates in plant tissues in response to a variety of abiotic and biotic stresses. γ-aminobutyric acid has a range of potential functions in plant responses to stress by regulating carbon and nitrogen balance, cytosolic pH and osmotic potential as well as protecting against oxidative stress damage, it is thought to be both a metabolite and signaling molecule [26]. Interestingly the application of both Gln and GABA stimulates callus formation in in vitro culture [27, 28].

The concentrations of the aromatic amino acids Tyr and Phe were lower at the graft interface in comparison to the surrounding woody scion and rootstock tissues. Phenylalanine and Tyr are important precursors in the formation of secondary metabolites, which accumulate in response to stresses such as wounding. The decrease in the concentration of Phe was associated with an increase in the activity of PAL, suggesting that Phe is converted into secondary metabolites at the graft interface.

Some changes in amino acid concentrations between the scion and rootstock stem and graft interface tissues of CS/CS grafts overlap with the metabolome reprogramming of wound induced callus in tomato [29] and induced by the transient over-expression of the *WOUND INDUCED DEDIFFERENTIATION1* (*WIND1*) in oilseed rape [30]. The APETALA2/ethylene response factor transcription factor WIND1 is a key regulator of wound-induced cellular reprogramming in Arabidopsis [31]. Tomato calli [29], the transient over-expression of *WIND1* [30], and the graft interface of grapevine are characterized by significantly higher concentrations of Gln and GABA, as well as increase of Ala, Val and Ile. This suggests that metabolome reprogramming in response to wound callus formation is similar between herbaceous and woody species, although the orthologue of *WIND1* in grapevine was not differentially expressed between the rootstock and graft interface tissues [21] and some amino acids show opposite responses. For example, Lys, Thr, and Phe accumulate in the callus cells of tomato relative to cotyledons [29], whereas they are in lower concentrations at the graft interface of grapevine in comparison to the surrounding woody stem tissues. Similarly, Pro, Ser, Asp and Gly accumulate in the callus tissues of tomato [29], but their concentration is not different between the stem and graft interface tissue of grapevine.

### Metabolite profiling in hetero-grafts suggests a genotype specific difference in the free amino acid and starch reserves formed for the winter months, and in the metabolome response of the graft interface

The comparison of the profile of primary metabolites between the different genotypes studied showed that CS had considerably higher levels of starch and Arg (and some other minor amino acids) than the two American rootstock species RG and 1103P. This could be due to differences in the formation of reserves in the autumn (both storage amino acids and their quantity) and/or differences in the mobilization of reserves from dormancy until 28 d after grafting. The higher starch and Arg content of the graft interface of CS/CS could be related to the higher concentration of starch and Arg in the rootstock (and scion) wood of CS than the other two genotypes, however, the lower water content suggests that these samples contained less callus tissue.

Some amino acids were specifically accumulated at the graft interface of the hetero-grafts in a manner which was unrelated to their rootstock tissue concentrations such as the high accumulation of branched chain amino acids (Ile, Leu and Val), Asn and Gln at the graft interface of CS/RG. Branched chain amino acids have an essential role in protein synthesis and the enzymes encoding their synthesis are generally highly expressed in young tissues [25]. Similarly Asp and Gln are key amino acids as they are precursors of other amino acids and metabolites. However, why branched chain amino acids, Asp and Gln are particularly accumulated at the graft interface of CS/RG and not the other two scion/rootstock combinations is not clear. The seasonal changes in free amino acid concentrations in the bark and wood of stems of poplar show that the concentration of many amino acids increases before or just after bud break (except Arg), when the metabolic activity of the wood increases [32]. This could suggest that the metabolic activity of the graft interface of CS/RG is higher than that of the two other scion/rootstock combinations, which could be possible as *V. riparia* are known to be early developing genotypes when grown un-grafted [33].

The concentration of Pro was higher in all tissues of the homo-graft, CS/CS, than the two hetero-grafted combinations, CS/RG and CS/1103P, suggesting that hetero-grafting reduced Pro accumulation both locally at the graft interface and at a short distance away in the scion. Proline is known to accumulate in plant tissues in response to abiotic stress, it has numerous functions in growth and development [34], and improves callus development in rice [28]. The increase in Pro in the homo-graft CS/CS could be related to an increase in the synthesis of Pro and/or a reduction of the flux of Pro to the formation of cell wall constituents compared to the hetero-grafts. The latter hypothesis is supported by the down-regulation of transcription of 8 proline rich proteins at the graft interface of CS/CS compared to CS/RG [4].

### Flavanols decrease at the graft interface of CS/CS

Generally, the concentration of flavanols (particularly epicatechin) decreases at the graft interface compared to the surrounding woody tissues, presumably the wood has a high concentration of flavanols, which gets diluted as the callus cells develop. Flavanol concentration, and particularly epicatechin, has been measured in a number of studies of graft incompatibility [15, 35, 36, 37, 38], but these studies often do not study the graft union itself or do not include homo-grafted controls so it is difficult to compare these results with the current work. However, one study on hetero-grafts of grapevine also reports a decrease in the concentration of epicatechin (but not catechin) in the graft interface relative to the surrounding woody tissues [15] suggesting that changes in this metabolite are typical of graft union formation in grapevine.

### Stilbenes accumulate at the graft interface 28 days after grafting

The increase in the abundance of transcripts encoding stilbene synthases at the graft interface of CS/CS 28 d after grafting was associated with an increase in the total concentration of stilbenes, which was primarily due to an increase in the concentration of the dimer *trans*-ε-viniferin (the major stilbene found in CS wood), and 3 trimers: *trans-* and *cis-*miyabenol C, and α-viniferin. Stilbenes have antioxidant capacities [39] as such these compounds could be as accumulated in response to the stress of grafting for their antioxidant properties. Wounding over-wintering canes of grapevine has also been shown to induce the expression of stilbene synthase transcripts and the accumulation of stilbenes [40], potentially suggesting that the accumulation of stilbenes is independent of callus cell formation. Stilbene synthesis is known to be stimulated by treatment with methyl jasmonate [41], a hormone typically synthesized in response to wounding, and transcripts encoding steps of the jasmonate biosynthesis pathway are also up-regulated at the graft interface of CS/CS 28 days after grafting [21].

Generally, the coupling of an increase in stilbene concentration and a decrease in flavonoid concentration is frequently observed in grapevine responses to stress including wounding [42].

### The profile of stilbenes in the rootstock wood is genotype-specific and certain stilbenes are differently accumulated at the graft interface of the different scion/rootstock combinations

The three genotypes of grapevine studied, CS, RG and 1103P, had different stilbene profiles in the rootstock wood tissues, genotypic variation in stilbene concentration has been previously reported in different cultivars of *V. vinifera * [43,44] and in the roots of different rootstock genotypes [45]. *Trans*-ε-viniferin is the main stilbene reported in canes for all cultivars [39, 43, 44], with amounts between 0.43–2.30 g kg^− 1^, which is consistent with our results.

The accumulation of stilbenes at the graft interface of the different scion/rootstock combinations is generally related to the concentration of stilbenes in the surrounding woody tissues; however, some stilbenes are specifically accumulated at the graft interface. Different genotypes of *Vitis spp.* show variation in the induction of stilbene synthesis in response to pathogens [46] and ultraviolet light treatments [47], suggesting that there is genotype-specific regulation of stilbene synthesis in response to stresses. The roles of individual stilbenes in plant defense and stress responses have yet to be identified. However, it is known that different stilbenes have different levels of toxicity to pathogens (with viniferins being more toxic stilbene monomers) and that glucosylation reduces the toxicity of these compounds [48].

## Conclusion

Grafting is a considerable stress to plants, resulting in the stimulation of wound responses and healing processes (namely the production of non-differentiated callus tissue and its differentiation into xylem and phloem). Metabolite profiling of primary metabolites at the graft interface of grapevine has revealed that the metabolome of the graft interface is reprogrammed to support callus cell proliferation, by the accumulation of Gln and GABA, and the reduction of Phe, Arg, Tyr and Lys concentrations. Globally, the concentration of flavanols decreased and the concentration of stilbenes increased at the graft interface in comparison to the surrounding tissues for all scion/rootstock combinations. There were genotypes specific differences in the profile of flavanols and stilbenes in the rootstock tissues, and although the concentration of these metabolites at the graft interface often reflected these genotype-specific differences, some specific metabolite accumulation patterns were observed. A small number of stilbenes showed scion/rootstock specific accumulation patterns; this could suggest that hetero-grafting two different genotypes together triggers specific changes in secondary metabolism and plant defense responses at the graft interface.

## Methods

### Plant material and grafting conditions

*Vitis vinifera* cv. ‘Cabernet-Sauvignon N’ (CS, clone 15; *Vitis* international variety catalogue number: 1929), *V. riparia* cv. ‘Riparia Gloire de Montpellier’ (RG, clone 1030; *Vitis* international variety catalogue number: 4824) and the *V. berlandieri* x *V. rupestris* hybrid cv. ‘1103 Paulsen’ (1103P, clone 198; *Vitis* international variety catalogue number: 9023) hardwood was collected from a vineyard in France in January (according to institutional guidelines) and stored as one meter long stems in a cold chamber (4 °C) until grafting in March. The identification of the plant material was done by the Centre de Ressources Biologiques de la Vigne, collection ampélographique de Vassal, Montpellier, France, by simple sequence repeats markers. No permissions were required to obtain this plant material. The scion CS was grafted onto RG (CS/RG) and 1103P (CS/1103P) as well as homo-grafted onto CS rootstocks (CS/CS); all these scion/rootstock combinations are highly compatible with grafting success rates of 87–95%. Dormant, stored stems (of approximately 0.8 to 1.2 cm in diameter) were taken out of the cold chamber 2 days before grafting and soaked in water at room temperature in order to rehydrate. One-bud cuttings were made for scions and two-node de-budded cuttings were made for rootstocks shortly before grafting. Bench mechanical omega grafting was performed on scion/rootstock pairs of approximately the same diameter. Grafts were briefly dipped into melted wax and placed into humid sawdust filled boxes for callusing at 28 °C for 28 d. Three pools of 15 graft interface zones (approximately 5 mm in length including both scion and rootstock tissues) were harvested and immediately snap frozen in liquid nitrogen. Two independent grafting experiments were done in the spring 2011 and 2012; some of this material was also used for previously published transcriptomic studies [4, 21] 

### Glucose, fructose, sucrose, starch and total protein measurements

The procedure of ethanolic extraction was followed as described by [49]. Glucose (Glc), fructose (Fru) and sucrose (Suc) were measured on ethanolic supernatant based on the protocol described in [50], and amino acids were measured as described below. Starch and total protein were measured on pellet in 100 mM NaOH as described by [49, 51]; assays were prepared in 96-well microplates and the absorbance of solutions was read at 340 and 595 nm respectively.

### Quantification of free amino acids

Free amino acids were measured after derivation with 6-aminoquinolyl-N-hydroxy-succinimidyl-carbamate (AccQ-Tag derivatization reagent, Waters) as described by [52]. Amino acids were quantified using an UltiMate 3000 ultra-HPLC system (Thermo Electron SAS) equipped with an FLD-3000 Fluorescence Detector (Thermo Electron SAS). Separation was performed on a AccQ•Tag Ultra column, 2.1 × 100 mm, 1.7 μm (Waters) at 37 °C with elution at 0.5 mL min^− 1^ (eluent A, sodium acetate buffer, 140 mM at pH 5.7; eluent B, acetonitrile; eluent C, water) according to the gradient described by [53]. Chromatograms corresponding to excitation at 250 nm and emission at 395 nm were recorded. To maintain consistent retention time and a stable baseline, a control was performed before each run of 18 samples. Chromeleon software, version 7.1 (Thermo Electron SAS), was used to calculate peak area. A standard set of 20 amino acids (alanine (Ala), arginine (Arg), aspartate (Asp), asparagine (Asn), cysteine (Cys), γ-aminobutyric acid (GABA), glycine (Gly), glutamate (Glu), glutamine (Gln), histidine (His), isoleucine (Ile), leucine (Leu), lysine (Lys), methionine (Met), phenylalanine (Phe), proline (Pro), serine (Ser), threonine (Thr), tyrosine (Tyr) and valine (Val)) purchased from Sigma was used after the control and in the middle of each run to quantify amino acid concentration.

### Enzyme extraction

For the measurement of NI and PAL activities, 500 mg of frozen tissue were extracted in 4 mL of 100 mM Tris-HCl buffer pH 8.8 containing 3 mM EDTA (ethylenediaminetetraacetic acid), 0.05% spermidine (w/v), 4 mM β-mercaptoethanol, 2 mM dithiothreitol, 0.1% triton X-100, a mixture of protease inhibitors (CompleteTM Protease Inhibitor Cocktail Tablets, Sigma), 1% (w/v) bovine serum albumin and 5% (w/v) polyvinylpolypyrrolidone. After centrifugation (20 min, 16,000 g at 4 °C), the protein extract was desalted using a PD-10 column (Sephadex resin G-25, PD-10 column, GE Healthcare) equilibrated with 25 mL of 100 mM Tris-HCl pH 8.8, 3 mM EDTA.

### Enzymes assays

The NI activity was assayed using a protocol from [54]. For the PAL activity, the assay contained 100 μL of enzymatic extract with 150 μL of phenylalanine 40 mM in 100 mM Tris-HCl pH 8.8, 3 mM EDTA. The production of cinnamic acid was followed and measured at 37 °C by the absorbance variation at 290 nm. The NI and PAL activities were quantified spectrophotometrically using a plate reader (Biotek) in 96-well polystyrene microplates.

### Quantification of phenolic compounds

One hundred mg of plant material were extracted in 1 mL of methanol in an ultrasound bath for 10 min. After centrifugation, the supernatants were filtered and diluted in milli Q water (1:1) for the analysis of flavanols and stilbenes. Flavanols were separated by HPLC on an Agilent SB-C18 RRHD column (2.1 x100mm, 1.8 μm) at 40 °C. The gradient was as follows: solvent A: 0.1% formic acid in milliQ water; solvent B: 0.1% formic acid in actonitrile (LC-MS grade); flow 0.4 mL min^− 1^; 0 min: 18% B; 3.5 min, 33% B; 6.5 min, 33% B; 12 min, 40% B; 13 min, 95% B; 16 min, 95% B; 16.5 min, 18% B. A calibration curve with catechin (Sigma), epicatechin (Sigma) and B2 (extrasynthese) in the range 0.04–50 mg L^− 1^ in 50% methanol was prepared. Detection was performed with a Triple Quadripole (6430 TQ, Agilent) operating under the following conditions: gas temperature: 350 °C, gas flow: 11 L min^− 1^, nebulizer 15 psi, voltage: 3000 V. Quantifier ions for each molecule were adapted from the literature [55]. Dimers and trimers were all expressed as equivalents of B2. Stilbenes were analyzed in the same system, but with a different gradient: solvent A: 0.1% formic acid in milliQ water; solvent B: 0.1% formic acid in actonitrile (LC-MS grade); flow 0.4 mL min^− 1^; 0 min: 10% B; 3 min, 20% B; 10 min, 33% B; 13 min, 33% B; 14 min, 50% B; 17 min, 95% B; 22 min, 95% B. A calibration curve with resveratrol, piceid, piceatannol, astringin, pallidol, parthenocissin, ε-viniferin, ω-viniferin, δ-viniferin, miyabenol C, hopeaphenol, isohopeaphenol, vitisin A and vitisinol C in the range 0.011–50 mg L^− 1^ was prepared. 1 μL was injected for each sample and standard. Detection was performed with a Triple Quadripole (6430 TQ, Agilent) operating under the following conditions: gas temperature: 350 °C, gas flow: 11 L min^− 1^, nebulizer 15 psi, voltage: 3000 V; mode of detection: MRM (transitions adapted) [56].

### Statistical analysis and data presentation

All data analysis and data presentation was done using the R environment [57] with the aid of the package pgirmess. Heatmaps were made with the package gplots [58], while PCA were made with ggplot2 and FactoMineR.

## Supplementary information

**Additional file 1:** **Table S1.** A comparison of water content (% H_2_O), phenylalanine ammonia lyase (PAL) activity, neutral invertase (NI) activity and the concentration of some metabolites in the scion, rootstock and graft interface of *Vitis vinifera* cv. Cabernet Sauvignon homo-grafts 28 d after grafting. When the conditions of an ANOVA were met (Shapiro and Barlett tests), means and *p* values are given, when conditions of an ANOVA were not met, median (indicated by stars) and *p* values of Kruskal-Wallis test are given. *P* values adjusted with Benjamini-Hochberg (BH) test. Letters indicate results of post hoc Tukey tests.

**Additional file 2:** **Table S2.** A comparison of water content (% H_2_O), phenylalanine ammonia lyase (PAL) activity, neutral invertase (NI) activity and the concentration of some metabolites in the rootstock wood of *Vitis vinifera* cv. Cabernet Sauvignon (CS) grafted with itself (CS/CS) and grafted with the rootstocks *V. berlandieri* x *V. rupestris* cv. 1103 Paulsen (CS/1103P) and *V. riparia* cv. Gloire de Montpellier (CS/RG) 28 d after grafting. When the conditions of an ANOVA were met (Shapiro and Barlett tests), means and *p* values are given, when conditions of an ANOVA were not met, median (indicated by stars) and *p* values of Kruskal-Wallis test are given. *P* values adjusted with Benjamini-Hochberg (BH) test. Letters indicate results of post hoc Tukey tests.

**Additional file 3:** **Table S3.** A comparison of water content (% H2O), phenylalanine ammonia lyase (PAL) activity, neutral invertase (NI) activity and the concentration of some metabolites at the graft interface of *Vitis vinifera* cv. Cabernet Sauvignon (CS) grafted with itself (CS/CS) and grafted with the rootstocks *V. berlandieri* x *V. rupestris* cv. 1103 Paulsen (CS/1103P) and *V. riparia* cv. Gloire de Montpellier (CS/RG) 28 d after grafting. When the conditions of an ANOVA were met (Shapiro and Barlett tests), means and *p* values are given, when conditions of an ANOVA were not met, median (indicated by stars) and *p* values of Kruskal-Wallis test are given. *P* values adjusted with Benjamini-Hochberg (BH) test. Letters indicate results of post hoc Tukey tests.

**Additional file 4:** **Table S4.** A comparison of the concentration of flavanols in the scion, rootstock and graft interface of *Vitis vinifera* cv. Cabernet Sauvignon homo-grafts 28 d after grafting. When the conditions of an ANOVA were met (Shapiro and Barlett tests), means and *p* values are given, when conditions of an ANOVA were not met, median (indicated by stars) and *p* values of Kruskal-Wallis test are given. *P* values adjusted with Benjamini-Hochberg (BH) test. Letters indicate results of post hoc Tukey tests.

**Additional file 5:** **Table S5.** A comparison of the concentration of flavanols in the rootstock wood of *Vitis vinifera* cv. Cabernet Sauvignon (CS) grafted with itself (CS/CS) and grafted with the rootstocks *V. berlandieri* x *V. rupestris* cv. 1103 Paulsen (CS/1103P) and *V. riparia* cv. Gloire de Montpellier (CS/RG) 28 d after grafting. When the conditions of an ANOVA were met (Shapiro and Barlett tests), means and *p* values are given, when conditions of an ANOVA were not met, median (indicated by stars) and *p* values of Kruskal-Wallis test are given. *P* values adjusted with Benjamini-Hochberg (BH) test. Letters indicate results of post hoc Tukey tests.

**Additional file 6:** **Table S6.** A comparison of the concentration of flavanols at the graft interface of *Vitis vinifera* cv. Cabernet Sauvignon (CS) grafted with itself (CS/CS) and grafted with the rootstocks *V. berlandieri* x *V. rupestris* cv. 1103 Paulsen (CS/1103P) and *V. riparia* cv. Gloire de Montpellier (CS/RG) 28 d after grafting. When the conditions of an ANOVA were met (Shapiro and Barlett tests), means and *p* values are given, when conditions of an ANOVA were not met, median (indicated by stars) and *p* values of Kruskal-Wallis test are given. *P* values adjusted with Benjamini-Hochberg (BH) test. Letters indicate results of post hoc Tukey tests.

**Additional file 7:** **Table S7.** A comparison of the concentration of stilbenes in the scion, rootstock and graft interface of *Vitis vinifera* cv. Cabernet Sauvignon homo-grafts 28 d after grafting. When the conditions of an ANOVA were met (Shapiro and Barlett tests), means and *p* values are given, when conditions of an ANOVA were not met, median (indicated by stars) and *p* values of Kruskal-Wallis test are given. *P* values adjusted with Benjamini-Hochberg (BH) test. Letters indicate results of post hoc Tukey tests.

**Additional file 8:** **Table S8.** A comparison of the concentration of stilbenes in the rootstock wood of *Vitis vinifera* cv. Cabernet Sauvignon (CS) grafted with itself (CS/CS) and grafted with the rootstocks *V. berlandieri* x *V. rupestris* cv. 1103 Paulsen (CS/1103P) and *V. riparia* cv. Gloire de Montpellier (CS/RG) 28 d after grafting. When the conditions of an ANOVA were met (Shapiro and Barlett tests), means and *p* values are given, when conditions of an ANOVA were not met, median (indicated by stars) and *p* values of Kruskal-Wallis test are given. *P* values adjusted with Benjamini-Hochberg (BH) test. Letters indicate results of post hoc Tukey tests.

**Additional file 9:** **Table S9.** A comparison of the concentration of stilbenes at the graft interface of *Vitis vinifera* cv. Cabernet Sauvignon (CS) grafted with itself (CS/CS) and grafted with the rootstocks *V. berlandieri* x *V. rupestris* cv. 1103 Paulsen (CS/1103P) and *V. riparia* cv. Gloire de Montpellier (CS/RG) 28 d after grafting. When the conditions of an ANOVA were met (Shapiro and Barlett tests), means and *p* values are given, when conditions of an ANOVA were not met, median (indicated by stars) and *p* values of Kruskal-Wallis test are given. *P* values adjusted with Benjamini-Hochberg (BH) test. Letters indicate results of post hoc Tukey tests.

## Data Availability

All data generated or analyzed during this study are included in this published article (and its additional files).

## Abbreviations

103P: *Vitis berlandieri* x *Vitis rupestris* cv. 1103 Paulsen
Ala: Alanine
ANOVA: Analysis of variance
Arg: Arginine
Asn: Asparagine
Asp: Aspartate
BH: Benjamini-Hochberg
CS: *Vitis vinifera* cv. Cabernet Sauvignon N
cv.: cultivar
Cys: Cysteine
EDTA: Ethylenediaminetetraacetic acid
Fru: Fructose
FW: Fresh weight
GABA: γ-aminobutyric acid
Glc: Glucose
Gln: Glutamine
Glu: Glutamate
Gly: Glycine
His: Histidine
HPLC: High-performance liquid chromatography
Ile: Isoleucine
Leu: Leucine
Lys: Lysine
Met: Methionine
NI: Neutral invertase
PAL: Phenylalanine ammonia lyase
PC: Principle component
PCA: Principle component analysis
Phe: Phenylalanine
Pro: Proline
RGM: *Vitis riparia* cv. Gloire de Montpellier
Ser: Serine
Suc: Sucrose
Thr: Threonine
Tris: Trisaminomethane
Tyr: Tyrosine
Val: Valine
WIND1: Wound Induced Dedifferentiation1
