# Supplementary material for: Metabolite profiling during graft union formation reveals the reprogramming of primary metabolism and the induction of stilbene synthesis at the graft interface in grapevine
Source: BMC Plant Biol. 2019 Dec 30;19:599. doi: 10.1186/s12870-019-2055-9 (PMC6937855; doi:10.1186/s12870-019-2055-9)
Supplement: Supplementary file 1 — Additional file 1: Table S1. A comparison of water content (% H2O), phenylalanine ammonia lyase (PAL) activity, neutral invertase (NI) activity and the concentration of some metabolites in the scion, rootstock and graft interface of Vitis vinifera cv. Cabernet Sauvignon homo-grafts 28 d after grafting. When the conditions of an ANOVA were met (Shapiro and Barlett tests), means and p values are given, when conditions of an ANOVA were not met, median (indicated by stars) and p values of Kruskal-Wallis test are given. P values adjusted with Benjamini-Hochberg (BH) test. Letters indicate results of post hoc Tukey tests. [file 12870_2019_2055_MOESM1_ESM.docx]

Additional file 1: Table S1. A comparison of water content (% H_2_O), phenylalanine ammonia lyase (PAL) activity, neutral invertase (NI) activity and the concentration of some metabolites in the scion, rootstock and graft interface of *Vitis vinifera* cv. Cabernet Sauvignon homo-grafts 28 d after grafting. When the conditions of an ANOVA were met (Shapiro and Barlett tests), means and *p* values are given, when conditions of an ANOVA were not met, median (indicated by stars) and *p* values of Kruskal-Wallis test are given. *P* values adjusted with Benjamini-Hochberg (BH) test. Letters indicate results of post hoc Tukey tests.

|  | Metabolite concentration | | | *p* values from statistical tests | | | |  |
| --- | --- | --- | --- | --- | --- | --- | --- | --- |
|  | Scion | Interface | Rootstock | Shapiro | Barlett | ANOVA | Kruskal-Wallis | BH adjusted *p* value |
| % H_2_O* | 53.0ab | 60.8a | 53.4b | 0.01 | 0.01 |  | 0.01 | 0.03 |
| Aspartate | 95.3 | 101.0 | 105.7 | 0.55 | 0.64 | 0.68 |  | 0.71 |
| Glutamate* | 196.8 | 160.2 | 183.7 | 0.01 | 0.19 |  | 0.68 | 0.71 |
| Serine | 27.7 | 37.1 | 31.1 | 0.09 | 0.38 | 0.38 |  | 0.49 |
| Asparagine* | 62.7 | 207.4 | 97.2 | 0.09 | 0.00 |  | 0.23 | 0.36 |
| Glycine | 3.9 | 5.1 | 5.3 | 0.52 | 0.30 | 0.55 |  | 0.66 |
| Glutamine | 336.2b | 751.1a | 321.3b | 0.73 | 0.86 | 0.00 |  | 0.00 |
| Histidine | 77.1a | 50.4b | 79.8a | 0.54 | 0.74 | 0.00 |  | 0.01 |
| Threonine | 103.5a | 56.0b | 102.1a | 0.98 | 0.15 | 0.01 |  | 0.03 |
| Arginine | 936.1a | 363.5b | 832.1a | 0.58 | 0.05 | 0.00 |  | 0.01 |
| Alanine | 17.1 | 20.9 | 16.2 | 0.69 | 0.51 | 0.35 |  | 0.49 |
| γ-aminobutyric acid | 31.2b | 55.4a | 33.4b | 0.98 | 0.11 | 0.02 |  | 0.04 |
| Proline | 52.2 | 50.6 | 53.6 | 0.99 | 0.30 | 0.96 |  | 0.96 |
| Tyrosine | 70.6a | 33.4b | 62.3a | 0.08 | 0.21 | 0.00 |  | 0.01 |
| Valine* | 22.3 | 28.3 | 25.8 | 0.01 | 0.53 |  | 0.18 | 0.29 |
| Methionine | 3.6 | 3.1 | 4.9 | 0.98 | 0.21 | 0.37 |  | 0.49 |
| Isoleucine | 18.4 | 29.3 | 19.7 | 0.48 | 0.29 | 0.03 |  | 0.06 |
| Leucine | 24.7 | 22.8 | 27.4 | 0.05 | 0.41 | 0.45 |  | 0.57 |
| Lysine* | 22.1a | 11.7b | 21.6ab | 0.62 | 0.04 |  | 0.01 | 0.03 |
| Phenylalanine | 9.5a | 5.5b | 9.2a | 0.51 | 0.39 | 0.02 |  | 0.04 |
| Total AA | 2081.7 | 1819.1 | 1836.9 | 0.08 | 0.61 | 0.67 |  | 0.71 |
| Proteins | 1.6b | 2.2a | 1.7b | 0.93 | 0.88 | 0.01 |  | 0.03 |
| PAL | 1.1b | 2.2a | 0.8b | 0.32 | 0.93 | 0.00 |  | 0.01 |
| NI* | 6.3 | 9.4 | 4.9 | 0.05 | 0.00 |  | 0.07 | 0.12 |
| Starch | 68.0b | 42.8c | 88.8a | 0.93 | 0.16 | 0.00 |  | 0.00 |
| Glucose | 1.7b | 2.8a | 2.0ab | 0.89 | 0.76 | 0.02 |  | 0.04 |
| Fructose | 1.5 | 1.8 | 1.7 | 0.60 | 0.10 | 0.57 |  | 0.66 |
| Sucrose | 6.8 | 5.5 | 6.4 | 0.85 | 0.92 | 0.31 |  | 0.46 |
| Total flavanols | 1445a | 1131b | 1336a | 0.35 | 0.21 | 0.00 |  | 0.01 |
| Total stilbenes | 1665b | 3152a | 1584b | 0.41 | 0.37 | 0.00 |  | 0.00 |

Amino acid (AA) concentrations given in pmol g^-1^ FW, sugars given µmol g^-1^ FW, proteins given in mg^-1^ g^-1^ FW, PAL and NI activity given in nmol min^-1^ g^-1^ FW, starch concentration given in equivalents of Glc (µmol Glc g^-1^ FW), total stilbenes and flavanols in mg kg^-1^ FW.
