# Supplementary material for: Metabolite profiling during graft union formation reveals the reprogramming of primary metabolism and the induction of stilbene synthesis at the graft interface in grapevine
Source: BMC Plant Biol. 2019 Dec 30;19:599. doi: 10.1186/s12870-019-2055-9 (PMC6937855; doi:10.1186/s12870-019-2055-9)
Supplement: Supplementary file 2 — Additional file 2: Table S2. A comparison of water content (% H2O), phenylalanine ammonia lyase (PAL) activity, neutral invertase (NI) activity and the concentration of some metabolites in the rootstock wood of Vitis vinifera cv. Cabernet Sauvignon (CS) grafted with itself (CS/CS) and grafted with the rootstocks V. berlandieri x V. rupestris cv. 1103 Paulsen (CS/1103P) and V. riparia cv. Gloire de Montpellier (CS/RG) 28 d after grafting. When the conditions of an ANOVA were met (Shapiro and Barlett tests), means and p values are given, when conditions of an ANOVA were not met, median (indicated by stars) and p values of Kruskal-Wallis test are given. P values adjusted with Benjamini-Hochberg (BH) test. Letters indicate results of post hoc Tukey tests. [file 12870_2019_2055_MOESM2_ESM.docx]

Additional file 2: Table S2. A comparison of water content (% H_2_O), phenylalanine ammonia lyase (PAL) activity, neutral invertase (NI) activity and the concentration of some metabolites in the rootstock wood of *Vitis vinifera* cv. Cabernet Sauvignon (CS) grafted with itself (CS/CS) and grafted with the rootstocks *V. berlandieri* x *V. rupestris* cv 1103 Paulsen (CS/1103P) and *V. riparia* cv Gloire de Montpellier (CS/RG) 28 d after grafting. When the conditions of an ANOVA were met (Shapiro and Barlett tests), means and p values are given, when conditions of an ANOVA were not met, median (indicated by stars) and *p* values of Kruskal-Wallis test are given. *P* values adjusted with Benjamini-Hochberg (BH) test. Letters indicate results of post hoc Tukey tests.

|  | Metabolite concentration | | | *p* values from statistical tests | | | |  |
| --- | --- | --- | --- | --- | --- | --- | --- | --- |
|  | CS/CS | CS/1103P | CS/RG | Shapiro | Barlett | ANOVA | Kruskal-Wallis | BH adjusted *p* value |
| % H_2_O | 53.2b | 53.7b | 58.5a | 0.11 | 0.10 | 0.01 |  | 0.02 |
| Aspartate | 105.7 | 122.8 | 116.9 | 0.24 | 0.72 | 0.53 |  | 0.67 |
| Glutamate | 168.2 | 163.8 | 149.9 | 0.11 | 0.60 | 0.67 |  | 0.74 |
| Serine | 31.1 | 25.5 | 29.1 | 0.10 | 0.71 | 0.69 |  | 0.74 |
| Asparagine* | 97.2b | 225.5a | 180.8ab | 0.01 | 0.02 |  | 0.01 | 0.02 |
| Glycine | 5.3 | 4.0 | 6.6 | 0.17 | 0.12 | 0.32 |  | 0.44 |
| Glutamine | 321.3 | 315.0 | 400.8 | 0.06 | 0.33 | 0.67 |  | 0.74 |
| Histidine | 79.8a | 47.4b | 52.5b | 0.83 | 0.73 | 0.00 |  | 0.01 |
| Threonine | 102.1a | 43.5b | 46.0b | 0.82 | 0.54 | 0.00 |  | 0.00 |
| Arginine | 832.1a | 199.5b | 217.4b | 0.91 | 0.92 | 0.00 |  | 0.00 |
| Alanine* | 16.5 | 16.3 | 12.4 | 0.01 | 0.08 |  | 0.76 | 0.79 |
| γ-aminobutyric acid | 33.4 | 27.5 | 33.3 | 0.23 | 0.75 | 0.61 |  | 0.73 |
| Proline* | 51.5a | 19.9b | 18.3b | 0.85 | 0.03 |  | 0.01 | 0.02 |
| Tyrosine | 62.3 | 42.1 | 46.4 | 0.15 | 0.09 | 0.03 |  | 0.06 |
| Valine | 27.7 | 30.0 | 31.5 | 0.07 | 0.41 | 0.87 |  | 0.87 |
| Methionine | 4.9a | 1.7b | 2.5b | 1.00 | 0.24 | 0.01 |  | 0.02 |
| Isoleucine* | 19.1 | 15.2 | 16.1 | 0.00 | 0.10 |  | 0.52 | 0.67 |
| Leucine | 27.4 | 20.4 | 25.7 | 0.61 | 0.07 | 0.15 |  | 0.24 |
| Lysine | 20.9 | 11.7 | 13.3 | 0.99 | 0.73 | 0.04 |  | 0.08 |
| Phenylalanine | 9.2a | 7.4ab | 4.7b | 0.64 | 0.45 | 0.02 |  | 0.04 |
| Total AA | 1836.9 | 1259.9 | 1456.5 | 0.13 | 0.60 | 0.18 |  | 0.27 |
| Proteins | 1.7 | 1.8 | 1.9 | 0.20 | 0.21 | 0.24 |  | 0.34 |
| PAL | 0.8 | 0.2 | 0.7 | 0.56 | 0.45 | 0.11 |  | 0.19 |
| NI | 4.9ab | 7.3a | 2.9b | 0.17 | 0.11 | 0.00 |  | 0.02 |
| Starch | 88.8a | 58.1b | 29.6c | 0.78 | 0.15 | 0.00 |  | 0.00 |
| Glucose | 2.0b | 3.1a | 3.2a | 0.73 | 0.72 | 0.01 |  | 0.02 |
| Fructose | 1.7a | 0.2b | 1.2b | 0.51 | 0.63 | 0.00 |  | 0.01 |
| Sucrose | 6.4a | 3.5b | 4.3b | 0.51 | 0.68 | 0.01 |  | 0.02 |
| Total Flavanols | 1263b | 2181a | 1485b | 0.3 | 0.1 | 0.0 |  | 0.00 |
| Total Stilbenes | 1584 | 2046 | 2344 | 0.2 | 1.6 | 0.0 |  | 0.09 |

Amino acid (AA) concentrations given in pmol g^-1^ FW, sugars given µmol g^-1^ FW, proteins given in mg^-1^ g^-1^ FW, PAL and NI activity given in nmol min^-1^ g^-1^ FW, starch concentration given in equivalents of Glc (µmol Glc g^-1^ FW), total stilbenes and flavanols in mg kg^-1^ FW.
