# Supplementary material for: Metabolite profiling during graft union formation reveals the reprogramming of primary metabolism and the induction of stilbene synthesis at the graft interface in grapevine
Source: BMC Plant Biol. 2019 Dec 30;19:599. doi: 10.1186/s12870-019-2055-9 (PMC6937855; doi:10.1186/s12870-019-2055-9)
Supplement: Supplementary file 3 — Additional file 3: Table S3. A comparison of water content (% H2O), phenylalanine ammonia lyase (PAL) activity, neutral invertase (NI) activity and the concentration of some metabolites at the graft interface of Vitis vinifera cv. Cabernet Sauvignon (CS) grafted with itself (CS/CS) and grafted with the rootstocks V. berlandieri x V. rupestris cv. 1103 Paulsen (CS/1103P) and V. riparia cv. Gloire de Montpellier (CS/RG) 28 d after grafting. When the conditions of an ANOVA were met (Shapiro and Barlett tests), means and p values are given, when conditions of an ANOVA were not met, median (indicated by stars) and p values of Kruskal-Wallis test are given. P values adjusted with Benjamini-Hochberg (BH) test. Letters indicate results of post hoc Tukey tests. [file 12870_2019_2055_MOESM3_ESM.docx]

Additional file 3: Table S3. A comparison of water content (% H_2_O), phenylalanine ammonia lyase (PAL) activity, neutral invertase (NI) activity and the concentration of some metabolites at the graft interface of *Vitis vinifera* cv. Cabernet Sauvignon (CS) grafted with itself (CS/CS) and grafted with the rootstocks *V. berlandieri* x *V. rupestris* cv 1103 Paulsen (CS/1103P) and *V. riparia* cv Gloire de Montpellier (CS/RG) 28 d after grafting. When the conditions of an ANOVA were met (Shapiro and Barlett tests), means and *p* values are given, when conditions of an ANOVA were not met, median (indicated by stars) and *p* values of Kruskal-Wallis test are given. *P* values adjusted with Benjamini-Hochberg (BH) test. Letters indicate results of post hoc Tukey tests.

|  | Metabolite concentration | | | *p* values from statistical tests | | | |  |
| --- | --- | --- | --- | --- | --- | --- | --- | --- |
|  | CS/CS | CS/1103P | CS/RG | Shapiro | Barlett | ANOVA | Kruskal-Wallis | BH adjusted *p* value |
| % H_2_O | 60.8b | 69.8b | 71.9a | 0.49 | 0.99 | 0.00 |  | 0.01 |
| Aspartate | 101.0b | 112.4b | 151.9a | 0.86 | 0.61 | 0.00 |  | 0.00 |
| Glutamate* | 160.2 | 149.2 | 190.4 | 0.00 | 0.01 |  | 0.15 | 0.21 |
| Serine | 37.1b | 42.2b | 65.7a | 0.90 | 0.40 | 0.00 |  | 0.01 |
| Asparagine | 186.3b | 354.4b | 554.5a | 0.84 | 0.92 | 0.00 |  | 0.00 |
| Glycine | 5.1b | 10.1a | 10.5a | 0.76 | 0.52 | 0.00 |  | 0.01 |
| Glutamine | 751.1b | 716.7b | 1029.7a | 0.45 | 0.78 | 0.02 |  | 0.03 |
| Histidine | 50.4 | 48.4 | 61.8 | 0.87 | 0.09 | 0.10 |  | 0.16 |
| Threonine | 56.0 | 49.9 | 66.1 | 0.99 | 0.28 | 0.16 |  | 0.22 |
| Arginine | 363.5a | 146.0b | 209.5ab | 0.78 | 0.19 | 0.01 |  | 0.02 |
| Alanine* | 20.3 | 33.2 | 32.5 | 0.01 | 0.01 |  | 0.04 | 0.07 |
| γ-aminobutyric acid | 55.4 | 55.6 | 74.1 | 0.96 | 0.51 | 0.11 |  | 0.16 |
| Proline | 50.6a | 28.3b | 22.7b | 0.16 | 0.61 | 0.00 |  | 0.00 |
| Tyrosine | 33.4 | 31.2 | 39.3 | 0.99 | 0.84 | 0.18 |  | 0.23 |
| Valine* | 28.3b | 45.0ab | 89.6a | 0.63 | 0.01 |  | 0.00 | 0.01 |
| Methionine | 2.5 | 1.7 | 3.2 | 0.20 | 0.17 | 0.29 |  | 0.33 |
| Isoleucine | 29.3b | 33.8b | 77.0a | 0.85 | 0.05 | 0.00 |  | 0.00 |
| Leucine | 22.8b | 22.7b | 46.8a | 0.41 | 0.08 | 0.00 |  | 0.00 |
| Lysine* | 11.7 | 7.4 | 10.4 | 0.53 | 0.03 |  | 0.09 | 0.16 |
| Phenylalanine | 5.5 | 6.0 | 5.9 | 0.68 | 0.78 | 0.79 |  | 0.82 |
| Total AA | 1819.1b | 1895.3b | 2709.7a | 0.49 | 0.70 | 0.00 |  | 0.01 |
| Proteins | 2.2 | 2.3 | 2.3 | 0.05 | 0.08 | 0.92 |  | 0.92 |
| PAL | 2.2 | 1.3 | 1.7 | 0.51 | 0.35 | 0.26 |  | 0.33 |
| NI | 10.2 | 10.4 | 5.0 | 0.06 | 0.25 | 0.09 |  | 0.16 |
| Starch | 42.8a | 24.6b | 12.5b | 0.09 | 0.00 | 0.00 |  | 0.00 |
| Glucose | 2.8 | 3.3 | 2.7 | 0.77 | 0.39 | 0.28 |  | 0.33 |
| Fructose | 1.8 | 1.3 | 1.5 | 0.14 | 0.50 | 0.57 |  | 0.61 |
| Sucrose | 5.5 | 4.3 | 4.4 | 0.29 | 0.47 | 0.34 |  | 0.38 |
| Total flavanols | 1150 | 1420 | 2822 | 0.9 | 0.1 | 0.0 |  | 0.06 |
| Total stilbenes | 3152a | 2099b | 2822a | 0.9 | 0.6 | 0.0 |  | 0.02 |

Amino acid (AA) concentrations given in pmol g^-1^ FW, sugars given µmol g^-1^ FW, proteins given in mg^-1^ g^-1^ FW, PAL and NI activity given in nmol min^-1^ g^-1^ FW, starch concentration given in equivalents of Glc (µmol Glc g^-1^FW), total stilbenes and flavanols in mg kg^-1^ FW.
