# Supplementary material for: Metabolite profiling during graft union formation reveals the reprogramming of primary metabolism and the induction of stilbene synthesis at the graft interface in grapevine
Source: BMC Plant Biol. 2019 Dec 30;19:599. doi: 10.1186/s12870-019-2055-9 (PMC6937855; doi:10.1186/s12870-019-2055-9)
Supplement: Supplementary file 4 — Additional file 4: Table S4. A comparison of the concentration of flavanols in the scion, rootstock and graft interface of Vitis vinifera cv. Cabernet Sauvignon homo-grafts 28 d after grafting. When the conditions of an ANOVA were met (Shapiro and Barlett tests), means and p values are given, when conditions of an ANOVA were not met, median (indicated by stars) and p values of Kruskal-Wallis test are given. P values adjusted with Benjamini-Hochberg (BH) test. Letters indicate results of post hoc Tukey tests. [file 12870_2019_2055_MOESM4_ESM.docx]

Additional file 4: Table S4. A comparison of the concentration of flavanols in the scion, rootstock and graft interface of *Vitis vinifera* cv. Cabernet Sauvignon homo-grafts 28 d after grafting. When the conditions of an ANOVA were met (Shapiro and Barlett tests), means and *p* values are given, when conditions of an ANOVA were not met, median (indicated by stars) and *p* values of Kruskal-Wallis test are given. *P* values adjusted with Benjamini-Hochberg (BH) test. Letters indicate results of post hoc Tukey tests.

|  | Flavanol concentration (mg kg^-1^) | | | *p* values from statistical tests | | | |  |
| --- | --- | --- | --- | --- | --- | --- | --- | --- |
|  | Scion | Interface | Rootstock | Shapiro | Bartlett | ANOVA | Kruskal-Wallis | BH adjusted *p* value |
| Catechin | 381 | 331 | 321 | 0.74 | 0.99 | 0.07 |  | 0.07 |
| Epicatechin | 456a | 270b | 447a | 0.96 | 0.42 | 0.00 |  | 0.00 |
| Epicatechin-gallate | 133 | 168 | 134 | 0.33 | 0.08 | 0.08 |  | 0.08 |
| Dimer B1 | 214a | 145c | 185b | 0.41 | 0.75 | 0.00 |  | 0.00 |
| Dimer B3 | 76a | 65b | 74ab | 0.43 | 0.75 | 0.02 |  | 0.02 |
| Dimer B4* | 30a | 24b | 28a | 0.00 | 0.02 |  | 0.00 | 0.00 |
| Dimer B2* | 47a | 32b | 46a | 0.03 | 0.02 |  | 0.00 | 0.00 |
