# Supplementary material for: Metabolite profiling during graft union formation reveals the reprogramming of primary metabolism and the induction of stilbene synthesis at the graft interface in grapevine
Source: BMC Plant Biol. 2019 Dec 30;19:599. doi: 10.1186/s12870-019-2055-9 (PMC6937855; doi:10.1186/s12870-019-2055-9)
Supplement: Supplementary file 5 — Additional file 5: Table S5. A comparison of the concentration of flavanols in the rootstock wood of Vitis vinifera cv. Cabernet Sauvignon (CS) grafted with itself (CS/CS) and grafted with the rootstocks V. berlandieri x V. rupestris cv. 1103 Paulsen (CS/1103P) and V. riparia cv. Gloire de Montpellier (CS/RG) 28 d after grafting. When the conditions of an ANOVA were met (Shapiro and Barlett tests), means and p values are given, when conditions of an ANOVA were not met, median (indicated by stars) and p values of Kruskal-Wallis test are given. P values adjusted with Benjamini-Hochberg (BH) test. Letters indicate results of post hoc Tukey tests. [file 12870_2019_2055_MOESM5_ESM.docx]

Additional file 5: Table S5. A comparison of the concentration of flavanols in the rootstock wood of *Vitis vinifera* cv. Cabernet Sauvignon (CS) grafted with itself (CS/CS) and grafted with the rootstocks *V. berlandieri* x *V. rupestris* cv 1103 Paulsen (CS/1103P) and *V. riparia* cv Gloire de Montpellier (CS/RG) 28 d after grafting. When the conditions of an ANOVA were met (Shapiro and Barlett tests), means and *p* values are given, when conditions of an ANOVA were not met, median (indicated by stars) and *p* values of Kruskal-Wallis test are given. *P* values adjusted with Benjamini-Hochberg (BH) test. Letters indicate results of post hoc Tukey tests.

|  | Flavanol concentration (mg kg^-1^) | | | *p* values from statistical tests | | | |  |
| --- | --- | --- | --- | --- | --- | --- | --- | --- |
|  | 1103P | CS | RG | Shapiro | Bartlett | ANOVA | Kruskal-Wallis | BH adjusted *p* value |
| Catechin | 778a | 321b | 843a | 0.98 | 0.82 | 0.00 |  | 0.00 |
| Epicatechin* | 620a | 447ab | 195b | 0.04 | 0.00 |  | 0.00 | 0.00 |
| Epicatechin-gallate * | 208a | 134ab | 83b | 0.49 | 0.01 |  | 0.00 | 0.00 |
| Dimer B1 | 311a | 185b | 193b | 0.58 | 0.05 | 0.00 |  | 0.00 |
| Dimer B3* | 124a | 74b | 79b | 0.11 | 0.04 |  | 0.00 | 0.00 |
| Dimer B4* | 42z | 30ab | 22b | 0.04 | 0.01 |  | 0.00 | 0.00 |
| Dimer B2* | 58a | 46a | 23b | 0.04 | 0.01 |  | 0.00 | 0.00 |
