# Supplementary material for: Metabolite profiling during graft union formation reveals the reprogramming of primary metabolism and the induction of stilbene synthesis at the graft interface in grapevine
Source: BMC Plant Biol. 2019 Dec 30;19:599. doi: 10.1186/s12870-019-2055-9 (PMC6937855; doi:10.1186/s12870-019-2055-9)
Supplement: Supplementary file 6 — Additional file 6: Table S6. A comparison of the concentration of flavanols at the graft interface of Vitis vinifera cv. Cabernet Sauvignon (CS) grafted with itself (CS/CS) and grafted with the rootstocks V. berlandieri x V. rupestris cv. 1103 Paulsen (CS/1103P) and V. riparia cv. Gloire de Montpellier (CS/RG) 28 d after grafting. When the conditions of an ANOVA were met (Shapiro and Barlett tests), means and p values are given, when conditions of an ANOVA were not met, median (indicated by stars) and p values of Kruskal-Wallis test are given. P values adjusted with Benjamini-Hochberg (BH) test. Letters indicate results of post hoc Tukey tests. [file 12870_2019_2055_MOESM6_ESM.docx]

Additional file 6: Table S6. A comparison of the concentration of flavanols at the graft interface of *Vitis vinifera* cv. Cabernet Sauvignon (CS) grafted with itself (CS/CS) and grafted with the rootstocks *V. berlandieri* x *V. rupestris* cv 1103 Paulsen (CS/1103P) and *V. riparia* cv Gloire de Montpellier (CS/RG) 28 d after grafting. When the conditions of an ANOVA were met (Shapiro and Barlett tests), means and *p* values are given, when conditions of an ANOVA were not met, median (indicated by stars) and *p* values of Kruskal-Wallis test are given. *P* values adjusted with Benjamini-Hochberg (BH) test. Letters indicate results of post hoc Tukey tests.

|  | Flavanol concentration (mg kg^-1^) | | | | | | *p* values from statistical tests | | | | | | |  | | |
| --- | --- | --- | --- | --- | --- | --- | --- | --- | --- | --- | --- | --- | --- | --- | --- | --- |
|  | 1103P | | CS | | RG | | Shapiro | | Bartlett | ANOVA | | Kruskal-Wallis | | BH adjusted *p* value | | |
| Catechin | 514a | 331b | | 587a | | 0.55 | | 0.66 | | | 0.00 | |  | | 0.00 |  |
| Epicatechin | 246a | 270a | | 145b | | 0.57 | | 0.12 | | | 0.01 | |  | | 0.02 |  |
| Epicatechin-gallate | 249a | 168b | | 133b | | 0.97 | | 0.34 | | | 0.00 | |  | | 0.00 |  |
| Dimer B1 | 166 | 145 | | 127 | | 0.98 | | 0.10 | | | 0.05 | |  | | 0.05 |  |
| Dimer B3* | 81 | 67 | | 73 | | 0.99 | | 0.05 | | |  | | 0.29 | | 0.29 |  |
| Dimer B4* | 25ab | 24a | | 17b | | 1.00 | | 0.01 | | |  | | 0.03 | | 0.04 |  |
| Dimer B2* | 31ab | 32a | | 23b | | 0.86 | | 0.04 | | |  | | 0.01 | | 0.02 |  |
