# Supplementary material for: Metabolite profiling during graft union formation reveals the reprogramming of primary metabolism and the induction of stilbene synthesis at the graft interface in grapevine
Source: BMC Plant Biol. 2019 Dec 30;19:599. doi: 10.1186/s12870-019-2055-9 (PMC6937855; doi:10.1186/s12870-019-2055-9)
Supplement: Supplementary file 7 — Additional file 7: Table S7. A comparison of the concentration of stilbenes in the scion, rootstock and graft interface of Vitis vinifera cv. Cabernet Sauvignon homo-grafts 28 d after grafting. When the conditions of an ANOVA were met (Shapiro and Barlett tests), means and p values are given, when conditions of an ANOVA were not met, median (indicated by stars) and p values of Kruskal-Wallis test are given. P values adjusted with Benjamini-Hochberg (BH) test. Letters indicate results of post hoc Tukey tests. [file 12870_2019_2055_MOESM7_ESM.docx]

Additional file 7: Table S7. A comparison of the concentration of stilbenes in the scion, rootstock and graft interface of *Vitis vinifera* cv. Cabernet Sauvignon homo-grafts 28 d after grafting. When the conditions of an ANOVA were met (Shapiro and Barlett tests), means and *p* values are given, when conditions of an ANOVA were not met, median (indicated by stars) and *p* values of Kruskal-Wallis test are given. *P* values adjusted with Benjamini-Hochberg (BH) test. Letters indicate results of post hoc Tukey tests.

|  | Stilbene concentration (mg kg^-1^) | | | p values from statistical tests | | | |  |
| --- | --- | --- | --- | --- | --- | --- | --- | --- |
|  | Scion | Interface | Rootstock | Shapiro | Bartlett | ANOVA | Kruskal-Wallis | BH adjusted p value |
| Monomers |  |  |  |  |  |  |  |  |
| *trans*-Astringin | 0.6b | 1.6a | 0.5b | 0.14 | 0.17 | 0.00 |  | 0.00 |
| *cis*-Astringin | 17.1a | 11.8b | 15.3a | 0.10 | 0.89 | 0.00 |  | 0.01 |
| *trans*-Piceid | 13.2ab | 15.2a | 9.7b | 0.25 | 0.91 | 0.00 |  | 0.01 |
| *cis*-Piceid | 0.5 | 0.8 | 0.5 | 0.54 | 0.77 | 0.03 |  | 0.05 |
| *trans*-Piceatannol* | 15.0ab | 19.7a | 8.5b | 0.00 | 0.00 |  | 0.02 | 0.03 |
| *cis*-Piceatannol* | 0.4 | 0.5 | 0.5 | 0.01 | 0.11 |  | 0.31 | 0.39 |
| *trans*-Resveratrol* | 169.9 | 188.6 | 138.3 | 0.35 | 0.01 |  | 0.15 | 0.25 |
| *cis*-Resveratrol | 1.2 | 1.3 | 1.5 | 0.10 | 0.12 | 0.74 |  | 0.81 |
| Dimers |  |  |  |  |  |  |  |  |
| Pallidol* | 16.7b | 82.0a | 12.7b | 0.12 | 0.02 |  | 0.00 | 0.01 |
| Parthenocissin A | 12.7b | 39.2a | 12.1b | 0.21 | 0.08 | 0.00 |  | 0.00 |
| *trans*-ε-Viniferin | 588.7b | 1137.4a | 539.0b | 0.19 | 0.06 | 0.00 |  | 0.00 |
| *cis*-ε-Viniferin* | 6.7 | 10.4 | 9.4 | 0.01 | 0.01 |  | 0.24 | 0.35 |
| *trans*-ω-Viniferin* | 7.4b | 68.6a | 8.3b | 0.08 | 0.00 |  | 0.00 | 0.01 |
| *trans*-δ-Viniferin | 24.6b | 57.0a | 25.5b | 0.24 | 0.12 | 0.00 |  | 0.00 |
| Ampelopsin A* | 13.2 | 12.2 | 13.6 | 0.01 | 0.05 |  | 0.70 | 0.80 |
| *trans*-Vitisinol C* | 1.0 | 1.5 | 1.1 | 0.67 | 0.01 |  | 0.25 | 0.35 |
| Trimers |  |  |  |  |  |  |  |  |
| *trans*-Miyabenol C | 32.6b | 103.3a | 35.9b | 0.55 | 0.35 | 0.00 |  | 0.01 |
| *cis*-Miyabenol C* | 91.3b | 339.0a | 58.1b | 0.04 | 0.58 |  | 0.00 | 0.01 |
| α-Viniferin* | 66.8b | 310.2a | 63.2b | 0.53 | 0.02 |  | 0.00 | 0.01 |
| Tetramers |  |  |  |  |  |  |  |  |
| Hopeaphenol | 317.5 | 327.0 | 334.5 | 0.68 | 0.12 | 0.94 |  | 0.98 |
| Isohopeaphenol | 147.4 | 189.6 | 107.4 | 0.08 | 0.33 | 0.27 |  | 0.36 |
| *trans-*Vitisin A | 9.2 | 8.4 | 7.8 | 0.21 | 0.29 | 0.69 |  | 0.80 |
| *trans-*Vitisin B | 128.2 | 189.2 | 146.8 | 0.80 | 0.12 | 0.16 |  | 0.25 |
