# Supplementary material for: Metabolite profiling during graft union formation reveals the reprogramming of primary metabolism and the induction of stilbene synthesis at the graft interface in grapevine
Source: BMC Plant Biol. 2019 Dec 30;19:599. doi: 10.1186/s12870-019-2055-9 (PMC6937855; doi:10.1186/s12870-019-2055-9)
Supplement: Supplementary file 8 — Additional file 8: Table S8. A comparison of the concentration of stilbenes in the rootstock wood of Vitis vinifera cv. Cabernet Sauvignon (CS) grafted with itself (CS/CS) and grafted with the rootstocks V. berlandieri x V. rupestris cv. 1103 Paulsen (CS/1103P) and V. riparia cv. Gloire de Montpellier (CS/RG) 28 d after grafting. When the conditions of an ANOVA were met (Shapiro and Barlett tests), means and p values are given, when conditions of an ANOVA were not met, median (indicated by stars) and p values of Kruskal-Wallis test are given. P values adjusted with Benjamini-Hochberg (BH) test. Letters indicate results of post hoc Tukey tests. [file 12870_2019_2055_MOESM8_ESM.docx]

Additional file 8: Table S8. A comparison of the concentration of stilbenes in the scion, rootstock and graft interface of *Vitis vinifera* cv. Cabernet Sauvignon homo-grafts 28 d after grafting. When the conditions of an ANOVA were met (Shapiro and Barlett tests), means and *p* values are given, when conditions of an ANOVA were not met, median (indicated by stars) and *p* values of Kruskal-Wallis test are given. *P* values adjusted with Benjamini-Hochberg (BH) test. Letters indicate results of post hoc Tukey tests.

|  | | Stilbene concentration (mg kg^-1^) | | | | | *p* values from statistical tests | | | | | | | |  | |  |
| --- | --- | --- | --- | --- | --- | --- | --- | --- | --- | --- | --- | --- | --- | --- | --- | --- | --- |
|  | | 1103P | CS | | RG | | Shapiro | | Bartlett | | ANOVA | | Kruskal-Wallis | | BH adjusted *p* value | |  |
| Monomers |  | | |  | |  | |  | |  | |  | |  | |  | |
| *trans*-Astringin | | 0.6 | 0.5 | | 0.8 | | 0.51 | | 0.84 | | 0.08 | |  | | 0.12 | |  |
| *cis*-Astringin* | | 0.9b | 14.3a | | 1.2ab | | 0.02 | | 0.00 | |  | | 0.00 | | 0.01 | |  |
| *trans*-Piceid* | | 24.2ab | 9.2b | | 56.7a | | 0.36 | | 0.02 | |  | | 0.00 | | 0.00 | |  |
| *cis*-Piceid | | 0.6 | 0.5 | | 0.6 | | 0.08 | | 0.63 | | 0.94 | |  | | 0.99 | |  |
| *trans*-Piceatannol | | 6.4 | 9.7 | | 5.7 | | 0.55 | | 0.14 | | 0.05 | |  | | 0.08 | |  |
| *cis*-Piceatannol | | 0.4 | 0.6 | | 0.4 | | 0.31 | | 0.89 | | 0.25 | |  | | 0.30 | |  |
| *trans*-Resveratrol | | 83.9b | 136.2a | | 121.2ab | | 0.26 | | 0.24 | | 0.01 | |  | | 0.03 | |  |
| *cis*-Resveratrol* | | 0.6 | 1.3 | | 0.7 | | 0.07 | | 0.01 | |  | | 0.11 | | 0.15 | |  |
| Dimers |  | | |  | |  | |  | |  | |  | |  | |  | |
| Pallidol | | 17.4b | 12.7b | | 27.2a | | 0.16 | | 0.42 | | 0.01 | |  | | 0.02 | |  |
| Parthenocissin A* | | 4.4 | 10.6 | | 4.5 | | 0.36 | | 0.01 | |  | | 0.04 | | 0.07 | |  |
| *trans*-ε-Viniferin | | 701.9ab | 519.4b | | 1347.3a | | 0.95 | | 0.27 | | 0.00 | |  | | 0.00 | |  |
| *cis*-ε-Viniferin* | | 9.4 | 9.4 | | 15.3 | | 0.04 | | 0.55 | |  | | 0.42 | | 0.46 | |  |
| *trans*-ω-Viniferin* | | 28.2a | 8.3b | | 19.0ab | | 0.06 | | 0.03 | |  | | 0.00 | | 0.01 | |  |
| *trans*-α-Viniferin | | 16.7 | 22.4 | | 27.0 | | 0.29 | | 0.32 | | 0.39 | |  | | 0.45 | |  |
| Ampelopsin A* | | 280.6a | 13.6b | | 10.6b | | 0.00 | | 0.00 | |  | | 0.00 | | 0.01 | |  |
| *trans*-Vitisinol C* | | 1.9ab | 1.1b | | 5.7a | | 0.15 | | 0.01 | |  | | 0.00 | | 0.00 | |  |
| Trimers |  | | |  | |  | |  | |  | |  | |  | |  | |
| *trans*-Miyabenol C* | | 5.1 | 30.6 | | 11.6 | | 0.01 | | 0.00 | |  | | 0.03 | | 0.06 | |  |
| *cis*-Miyabenol C* | | 20.9 | 58.1 | | 19.7 | | 0.01 | | 0.00 | |  | | 0.14 | | 0.18 | |  |
| α-Viniferin* | | 10.6 | 63.2 | | 34.0 | | 0.10 | | 0.00 | |  | | 0.08 | | 0.12 | |  |
| Tetramers |  | | |  | |  | |  | |  | |  | |  | |  | |
| Hopeaphenol* | | 112.1b | 343.4a | | 276.3a | | 0.18 | | 0.00 | |  | | 0.00 | | 0.01 | |  |
| Isohopeaphenol* | | 25.6 | 93.7 | | 220.8 | | 0.47 | | 0.00 | |  | | 0.03 | | 0.06 | |  |
| *trans*-Vitisin A* | | 47.1a | 8.2b | | 23.2ab | | 0.00 | | 0.00 | |  | | 0.00 | | 0.00 | |  |
| *trans*-Vitisin B* | | 413.9a | 154.7b | | 203.3ab | | 0.00 | | 0.00 | |  | | 0.00 | | 0.01 | |  |
