# Supplementary material for: Metabolite profiling during graft union formation reveals the reprogramming of primary metabolism and the induction of stilbene synthesis at the graft interface in grapevine
Source: BMC Plant Biol. 2019 Dec 30;19:599. doi: 10.1186/s12870-019-2055-9 (PMC6937855; doi:10.1186/s12870-019-2055-9)
Supplement: Supplementary file 9 — Additional file 9: Table S9. A comparison of the concentration of stilbenes at the graft interface of Vitis vinifera cv. Cabernet Sauvignon (CS) grafted with itself (CS/CS) and grafted with the rootstocks V. berlandieri x V. rupestris cv. 1103 Paulsen (CS/1103P) and V. riparia cv. Gloire de Montpellier (CS/RG) 28 d after grafting. When the conditions of an ANOVA were met (Shapiro and Barlett tests), means and p values are given, when conditions of an ANOVA were not met, median (indicated by stars) and p values of Kruskal-Wallis test are given. P values adjusted with Benjamini-Hochberg (BH) test. Letters indicate results of post hoc Tukey tests. [file 12870_2019_2055_MOESM9_ESM.docx]

Additional file 9: Table S9. A comparison of the concentration of stilbenes at the graft interface of *Vitis vinifera* cv. Cabernet Sauvignon (CS) grafted with itself (CS/CS) and grafted with the rootstocks *V. berlandieri* x *V. rupestris* cv 1103 Paulsen (CS/1103P) and *V. riparia* cv Gloire de Montpellier (CS/RG) 28 d after grafting. When the conditions of an ANOVA were met (Shapiro and Barlett tests), means and *p* values are given, when conditions of an ANOVA were not met, median (indicated by stars) and *p* values of Kruskal-Wallis test are given. *P* values adjusted with Benjamini-Hochberg (BH) test. Letters indicate results of post hoc Tukey tests.

|  | Stilbene concentration (mg kg^-1^) | | | | | *p* values from statistical tests | | | | | | | |  | |  |
| --- | --- | --- | --- | --- | --- | --- | --- | --- | --- | --- | --- | --- | --- | --- | --- | --- |
|  | 1103P | CS | | RG | | Shapiro | | Bartlett | | ANOVA | | Kruskal-Wallis | | BH adjusted *p* value | |  |
| Monomers |  | |  | |  | |  | |  | |  | |  | |  | |
| *trans*-Astringin | 2.6a | 1.6b | | 3.4a | | 0.32 | | 0.43 | | 0.00 | |  | | 0.00 | |  |
| *cis*-Astringin* | 4.5b | 11.6a | | 4.4b | | 0.03 | | 0.01 | |  | | 0.00 | | 0.01 | |  |
| *trans*-Piceid* | 42.5ab | 15.2b | | 72.2a | | 0.60 | | 0.04 | |  | | 0.00 | | 0.00 | |  |
| *cis*-Piceid | 0.9b | 0.8b | | 1.3a | | 0.16 | | 0.25 | | 0.01 | |  | | 0.02 | |  |
| *trans*-Piceatannol* | 8.6b | 19.7a | | 16.8ab | | 0.00 | | 0.01 | |  | | 0.03 | | 0.03 | |  |
| *cis*-Piceatannol* | 0.4 | 0.5 | | 0.6 | | 0.10 | | 0.02 | |  | | 0.50 | | 0.50 | |  |
| *trans*-Resveratrol | 143.4 | 217.9 | | 164.6 | | 0.76 | | 0.15 | | 0.22 | |  | | 0.24 | |  |
| *cis*-Resveratrol | 0.9 | 1.3 | | 1.1 | | 0.41 | | 0.96 | | 0.40 | |  | | 0.41 | |  |
| Dimers |  | |  | |  | |  | |  | |  | |  | |  | |
| Pallidol | 48.0b | 79.5a | | 67.7ab | | 0.38 | | 0.29 | | 0.00 | |  | | 0.01 | |  |
| Parthenocissin A | 21.2b | 39.2a | | 23.7b | | 0.31 | | 0.13 | | 0.00 | |  | | 0.00 | |  |
| *trans*-ε-Viniferin | 725.4b | 1137.4a | | 1086.3a | | 0.35 | | 0.17 | | 0.01 | |  | | 0.02 | |  |
| *cis*-ε-Viniferin* | 6.8b | 10.4ab | | 12.4a | | 0.92 | | 0.04 | |  | | 0.01 | | 0.02 | |  |
| *trans*-ω-Viniferin | 34.4b | 64.4a | | 38.6b | | 0.42 | | 0.08 | | 0.00 | |  | | 0.00 | |  |
| *trans*-δ-Viniferin | 39.0b | 57.0a | | 52.9ab | | 0.70 | | 0.57 | | 0.02 | |  | | 0.02 | |  |
| Ampelopsin A* | 101.5a | 12.2ab | | 8.4b | | 0.15 | | 0.00 | |  | | 0.00 | | 0.00 | |  |
| *trans*-Vitisinol C | 1.1b | 1.5b | | 2.4a | | 0.99 | | 0.19 | | 0.00 | |  | | 0.00 | |  |
| Trimers |  | |  | |  | |  | |  | |  | |  | |  | |
| *trans*-Miyabenol C* | 54.9b | 99.2a | | 61.7ab | | 0.04 | | 0.00 | |  | | 0.01 | | 0.02 | |  |
| *cis*-Miyabenol C | 153.5b | 327.6a | | 173.8b | | 0.17 | | 0.91 | | 0.00 | |  | | 0.00 | |  |
| α-Viniferin* | 141.8b | 310.2a | | 197.3ab | | 0.19 | | 0.00 | |  | | 0.01 | | 0.02 | |  |
| Tetramers |  | |  | |  | |  | |  | |  | |  | |  | |
| Hopeaphenol | 182.9b | 327.0a | | 367.4a | | 0.89 | | 0.10 | | 0.00 | |  | | 0.01 | |  |
| Isohopeaphenol | 142.4b | 189.6b | | 328.1a | | 1.00 | | 0.42 | | 0.00 | |  | | 0.00 | |  |
| *trans*-Vitisin A | 19.7a | 8.4b | | 13.4ab | | 0.34 | | 0.20 | | 0.02 | |  | | 0.02 | |  |
| *trans*-Vitisin B | 214.1 | 189.2 | | 125.4 | | 0.28 | | 0.16 | | 0.10 | |  | | 0.11 | |  |
